# Supplementary material for: The impact of pulse oximetry and Integrated Management of Childhood Illness (IMCI) training on antibiotic prescribing practices in rural Malawi: A mixed-methods study
Source: PLoS One. 2020 Nov 19;15(11):e0242440. doi: 10.1371/journal.pone.0242440 (PMC7676725; doi:10.1371/journal.pone.0242440)
Supplement: S1 Text — (DOCX) [file pone.0242440.s001.docx]

**RESPIRATORY ILLNESS QUALITATIVE INTERVIEW GUIDE FOR GAIA CLINIC STAFF**

IMCI Continued Education Course – ALL CLINIC STAFF

1. How do you feel that the IMCI training course influenced your decision to diagnose pediatric patients?

2. Do you think that the IMCI training course was beneficial to your skills as a clinical provider? If so, how?

3a. FOR NURSES: Based on the IMCI training, do you feel confident in being able to diagnose pediatric patients by yourself? Or would you still prefer the clinical officer to have this responsibility?

3b. FOR CLINICAL OFFICERS: Based on the IMCI training, do you believe that the nurses are able to diagnose pediatric patients independently?

4. What was easy or difficult about participating in the IMCI training course?

5. Do you believe that this course should be continued for the GAIA staff in future years?

Pulse Oximeters – ONLY FOR CLINIC STAFF AT MHCS RECEIVING PULSE OXIMETERS

1. How do you feel that the pulse oximeter influenced your diagnosis for pediatric patients with non-malarial fever?

2. What was easy or difficult about using the pulse oximeters in the clinics?

3. Do you believe that using the pulse oximeter impacted how frequently you prescribed antibiotics to pediatric patients with non-malarial fever? If so, how?

4. Do you believe that pulse oximeters should continue to be used by clinical providers in the GAIA mobile health clinics?

5. Are there any other tools either in addition to or that could replace the IMCI course or pulse oximeter that you would find useful for diagnosing non-malarial fever?
